# Iron oxide nanoparticles in leukemia: design, diagnostic applications, and therapeutic strategies

**DOI:** 10.1186/s43046-025-00301-2

**Published:** 2025-06-03

**Authors:** Henry Fenekansi Kiwumulo, Haruna Muwonge, Michael Lubwama, Charles Ibingira, John Baptist Kirabira, Robert Tamale Ssekitoleko, Stephen Evans

**Affiliations:** 1 Makerere University, Kampala, Uganda; 2 Department of Molecular & Nanoscale Physics, University of Leeds, Leeds, UK

**Keywords:** Leukemia, Iron oxide nanoparticles, Diagnostic, Therapeutic, Hydrophilic design, Hydrophobic design, Alternating magnetic field

## Abstract

Leukemia, a heterogeneous group of hematologic malignancies, poses significant challenges in terms of early diagnosis and effective treatment. Recent advancements in nanotechnology have paved the way for innovative approaches in leukemia management, with a particular focus on IONPs. This review paper explores the diverse designs of IONPs and their multifaceted applications in the diagnosis and treatment of leukemia. Focused discussions on the synergistic combination of IONPs with conventional chemotherapy, targeted drug delivery, and hyperthermia-based approaches provide insights into the evolving landscape of IONP-mediated leukemia therapy. The role of IONPs in overcoming drug resistance mechanisms and minimizing off-target effects is critically evaluated. The later review section provides an overview of the unique physical, chemical, and magnetic properties of IONPs, emphasizing their biocompatibility, tunable magnetic properties, and surface functionalization capabilities. The review finally addresses the challenges and prospects associated with the clinical translation of IONP-based diagnostic approaches. By addressing the challenges and opportunities in this burgeoning field, this paper aims to guide future research endeavors toward the development of effective and personalized nanotherapeutics for leukemia patients.

## Introduction

Nanomedicine has emerged as a transformative field in biomedical science, enabling the development of novel diagnostic and therapeutic tools that operate at the molecular and cellular levels [95]. Among the various nanomaterials under investigation, Iron Oxide Nanoparticles (IONPs) have garnered significant interest due to their unique magnetic properties, biocompatibility, and tunable surface chemistry. These properties allow IONPs to serve as multifunctional agents in targeted drug delivery, magnetic resonance imaging (MRI), hyperthermia therapy, and biosensing applications—especially in cancer diagnostics and treatment [71, 134]. Iron oxides such as magnetite (Fe₃O₄), maghemite (γ-Fe₂O₃), and hematite (α-Fe₂O₃) exhibit superparamagnetic behavior at the nanoscale, allowing for controlled manipulation under external magnetic fields without residual magnetization. These characteristics are particularly useful in cancer therapy, where selective targeting of tumor cells while sparing normal tissue remains a central challenge [77]. The synthesis and surface functionalization of IONPs further enable their adaptation for hydrophilic or hydrophobic drug encapsulation, receptor-mediated targeting, and real-time imaging, making them highly adaptable platforms for theranostics [134].

Within the context of cancer, the use of iron oxides, particularly IONPs, has gained significant attention [21, 58, 76, 93, 106, 120]. Leukemia—a group of hematological malignancies involving the uncontrolled proliferation of blood-forming cells—presents distinct therapeutic challenges due to its systemic nature, frequent relapses, and the development of drug resistance [116]. Conventional chemotherapy, while initially effective, is often associated with systemic toxicity, limited selectivity, and multidrug resistance, necessitating novel approaches that enhance therapeutic specificity and minimize collateral damage [124]. Leukemia, characterized by the abnormal proliferation of blood cells, presents a complex and heterogeneous challenge in the landscape of cancer [78, 104, 122]. The nature of leukemia, its subtypes, and the mechanisms driving its progression necessitate innovative therapeutic approaches. Despite advancements in leukemia treatment, the status reveals persistent challenges, including the risk of relapse, side effects of conventional therapies, and the development of drug resistance [23, 138]. Complications associated with leukemia treatment further underscore the need for novel and targeted therapeutic strategies. These complications encompass hematological toxicity, immunosuppression, and long-term adverse effects, emphasizing the urgency to develop therapies that are both efficacious and minimally detrimental to normal cells [51].

In this context, IONPs offer a promising solution for leukemia treatment. Their magnetic properties allow for guided delivery and accumulation in leukemic tissues, and their surfaces can be functionalized with targeting ligands, antibodies, or siRNA to enable personalized and precision-based therapy [3, 46, 50, 75, 76, 120, 147]. Iron oxides exist in various forms, each possessing unique properties that impact their behavior in biological systems. Understanding these different forms, including magnetite (Fe_3_O_4_), maghemite (γ-Fe_2_O_3_), and hematite (α-Fe_2_O_3_), is essential for tailoring IONPs to specific biomedical applications [42]. Furthermore, the special characteristics inherent to iron oxides, such as their magnetic responsiveness, biocompatibility, and ease of surface functionalization, contribute to their exceptional suitability for biomedical purposes [66]. The efficiency of IONPs is influenced by numerous factors, ranging from size, shape, and surface chemistry to the method of synthesis [79]. Unraveling these intricacies is crucial for optimizing the design of IONPs for enhanced performance in various biomedical applications, including cancer treatment [31, 136, 145]. Additionally, the ability of IONPs to act as carriers for small molecules and nucleic acids provides a versatile platform for combination therapies that can address multiple disease mechanisms simultaneously [88].

Despite the extensive exploration of IONPs in solid tumors, their application in hematological malignancies such as leukemia is relatively underrepresented in the literature. This review bridges that gap by focusing on the current advances in the design of IONPs tailored for leukemia applications, covering diagnostic, therapeutic, and combined theranostic strategies. We systematically examine the hydrophilic and hydrophobic modifications that influence drug delivery efficiency, imaging performance, and biocompatibility. Moreover, we explore how IONPs can overcome current treatment limitations such as drug resistance, poor bioavailability, and off-target effects. In the rapidly evolving landscape of nanomedicine, understanding the role of IONPs in hematologic malignancies is crucial for the development of next-generation therapies. This review aims to synthesize the current knowledge, identify challenges, and propose future directions for clinical translation of IONPs in leukemia diagnostics and therapy. This paper examines the applications of iron oxides in cancer treatment, highlighting their roles in imaging, drug delivery, and therapeutic hyperthermia. This sets the stage for a more in-depth exploration of their application in leukemia treatment. The review comprehensively explores the diverse facets of IONPs, ranging from their distinct forms and special characteristics to factors influencing their efficiency and their applications in cancer treatment, specifically focusing on leukemia [33, 130]. Motivated by the imperative to address the limitations of current leukemia treatments, this review focuses on the essence of studying leukemia treatment using different designs of IONPs. By critically evaluating the potential of IONPs in improving the specificity, efficacy, and safety of leukemia therapy, this review aims to provide insights that guide future research endeavors and contribute to the evolution of precision medicine in the context of hematological malignancies.

## Leukemia diagnosis and treatment using IONPs

The integration of IONPs in leukemia diagnosis and treatment provides a multifaceted and innovative approach. From small molecule drug delivery to antibody therapy and gene therapy, these nanoparticles play a pivotal role in advancing precision medicine for leukemia patients. Additionally, their applications in photothermal therapy and imaging underscore their potential to revolutionize the landscape of leukemia management. In this section, we will explore the design features (hydrophilic and hydrophobic) of each coating material and how they contribute to the diagnosis or treatment of leukemia using IONPs as outlined in Table 1.

**Table 1:** Hydrophilic and hydrophobic surface functionalization of IONPs in various cancer applications

Hydrophilic contribution	Hydrophobic contribution	Drug	Cancer cells used	Intended purpose	Reference
Poly(ethylene oxide) (PEO)	Oleic acid, Poly(propylene oxide) (PPO)	DOX	MCF-7 and PC3	Small molecule drug therapy	[57]
Anti-synthetic peptide antibody (EGFRvIIIAb)	Carboxyl group (COOH)	–	Glioblastoma cell	Antibody therapy	[48]
(γ-Mercaptopropyl)trimethoxysilane (MPS), Poly(ethylene glycol) dimethacrylate (PGD), silica	Mercaptopropyl group, Thiol-modified KH1 C12 aptamer, mercaptopropyl group	–	Human leukemic cancer cells (HL-60)	Imaging and diagnostics	[49]
Poly(carboxybetaine methacrylate) (pCBMA) Brushes, Dual-Antibody Interface (EpCAM and N-cadherin)	TEOS, carboxyl group	–	Circulating tumor cells (CTCs)	Imaging and diagnostics	[128, 131]
Magnetic mesoporous silica nanoparticles, antibody of epithelial cell adhesion molecule (EpCAM), CTAB (Cetyltrimethylammonium bromide), NH4OH (Ammonium hydroxide)	TEOS (Tetraethylorthosilicate), DMF (Dimethylformamide)	–	Breast cancer cells (MCF-7) and CTCs	Imaging and diagnostics	[19]
PEG	MoS2 (MS) Flakes	–	Hela and HepG2 cells	Photothermal therapy	[141]
PEG	Perfluorooctylbromide (PFOB), Poly(lactic acid) (PLA)	–	Human tumor xenograft models	Photothermal therapy	[63]
Dextran, folate	Retinoic acid	DOX	MCF-7 and MDA-MB-468 cells	Small molecule drug therapy	[127]
Poly(γ-glutamic acid) (PGA)	Distearyl γ-glutamate (DSGA), Oleic acid	Paclitaxel (PTX)		Small molecule drug therapy	[53]
PEG	PLGA	DOX	NIH 3 T3 fibroblast cells, breast cancer (MCF-7) cells	Small molecule drug therapy	[73]
PEG	Dimethyl sulfoxide (DMSO)	6-mercaptopurine (MP)	leukemic WEHI-3B	Small molecule drug therapy	[32]
Silica	Tetraethoxysilane (TEOS)	Cytarabine	HL60 cells	Small molecule drug therapy	[109]
Porous silica	CTAB (Cetyltrimethylammonium bromide)	Papain	HeLa cancer cells	Small molecule drug therapy	[91]
Dextran	Ferrous chloride tetrahydrate and ferric chloride hexahydrate	5-Fluorouracil (5-FU)	Caco-2 cells	Small molecule drug therapy	[9]
Dextran, folate	DMSO (Dimethyl sulfoxide)	Camptothecin (CPT)	Prostate cancer cells	Small molecule drug therapy	[5]
Silica, PVA, and human IgG1 sortilin antibody (SORT)	TEOS (Tetraethylorthosilicate), CPTMS (3-Chloropropyl Trimethoxysilane),CPS (Chloropropylsilane)	Taxotere (TXT)	Caov-4 ovarian cancer cells	Antibody therapy	[121]
Dextran, anti-HER2 antibody (trastuzumab)	Carboxyl group (COOH)	–	Breast cancer cells (AU565), human gastric cancer cells (NUGC-4), human ovarian cancer cells (SK-OV-3)	Antibody therapy	[60]
MXD3 siRNA, anti-CD22 antibodies	Succinimidyl ester group	–	preB ALL	Gene therapy	[108]
Galactose (Gal) and siRNA	PEI	–	Hepa1–6 cells	Gene therapy	[140]
Chitosan, PEG, and siRNA	Catechol Moiety, PEI	–	Glioma cells	Gene therapy	[27]

## Leukemia treatment using small molecule drug delivery approach

In recent years, the small molecule drug delivery approach has emerged as a promising strategy for improving the efficacy and specificity of leukemia treatment. Various coating materials have been explored to encapsulate small molecules, enhancing their delivery to leukemia cells while minimizing off-target effects. This section discusses the application of lipid coatings, silica shells, and dextran coatings in the context of small molecule drug delivery for leukemia therapy.

### Lipid coatings

The synergistic effects of oleic acid and PEG coatings on IONPs play a vital role in optimizing their performance in small molecule drug delivery for leukemia treatment. These lipid coatings not only confer stability and biocompatibility to the nanoparticles but also enable precise targeting, ensuring that therapeutic payloads reach their intended destination with enhanced efficacy. As a result, the strategic use of lipid coated IONPs represents a promising avenue in the ongoing pursuit of more effective and targeted therapies for leukemia.

#### Oleic acid coating

**Fig. 1:**
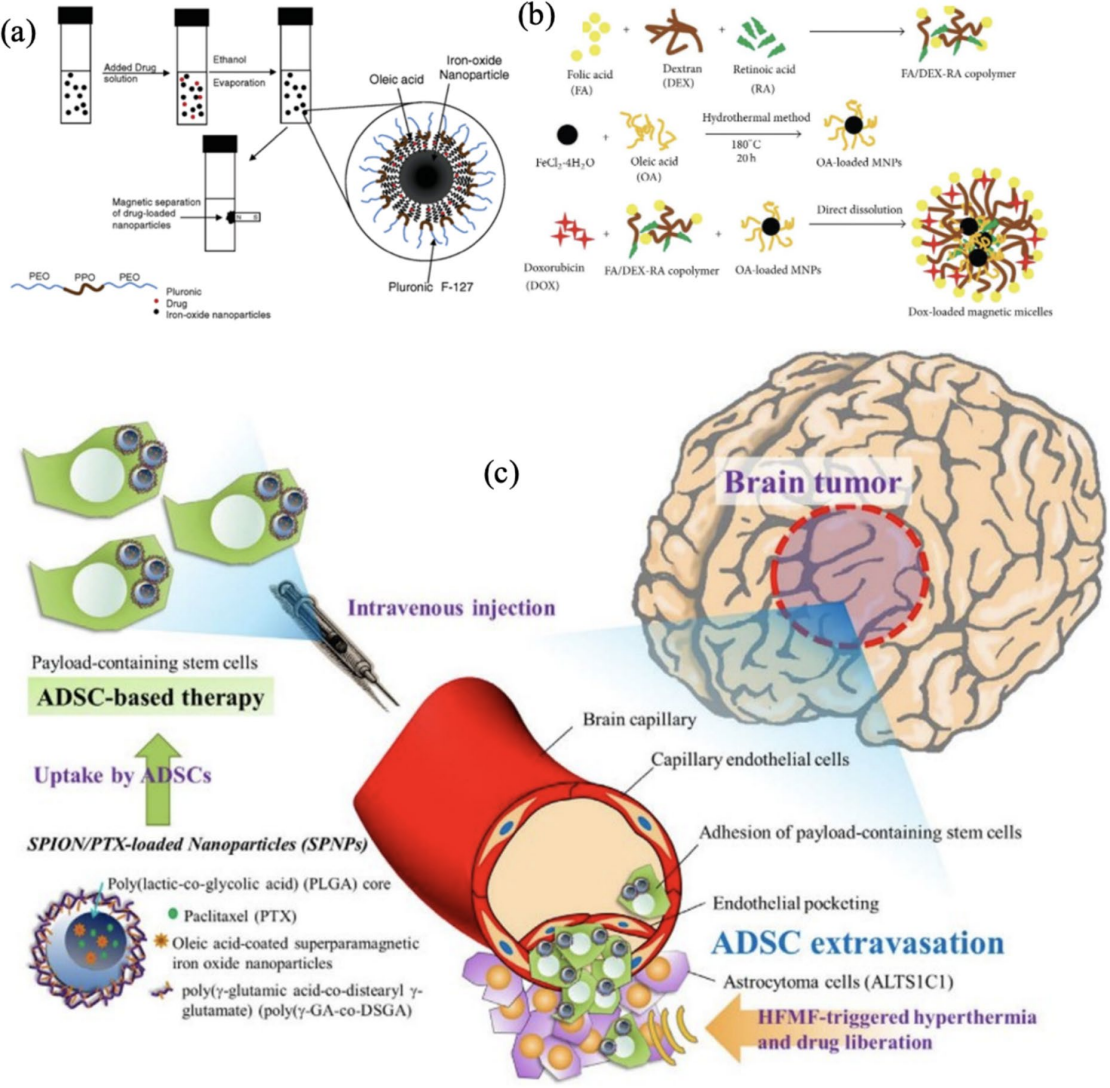
**a** diagram illustrating the formulation of IONPs and the procedure for loading drugs using Oleic acid [57]. **b** Formation of Oleic acid coated IONP as magnetic micelles [127]. **c** Illustration depicting the ADSC-facilitated transportation of Oleic acid coated IONPs to target brain tumors, aiming for a combined therapeutic approach for orthotopic astrocytoma [53]

Some groups have utilized oleic acid (OA) as a coating for the IONPs to improve the particle stability and biocompatibility [53, 57, 127]. Jain’s group developed a water-dispersible OA-Pluronic-coated IONP formulation for easy loading of doxorubicin (DOX) (Fig. 1a). Pluronic is a hydrophilic polymer that anchors at the OA-water interface, providing aqueous dispersity to the formulation [21]. The hydrophilic nature of Pluronic helps in the dispersion of the nanoparticles in water. OA forms a hydrophobic shell surrounding IONPs and providing for the DOX drug. The hydrophobic interaction between OA and the drug contributes to drug loading in the OA shell. Jain’s group claimed to provide the first report using their OA coated IONP formulation. The group showed sustained DOX release over 2 weeks in vitro, demonstrated sustained intracellular drug retention, and exhibited a dose-dependent antiproliferative effect in MCF-7 and PC3 cancer cell lines. Varshosaz’s group self-assembled an amphiphilic copolymer of folate-conjugated dextran/retinoic acid (FA/DEX-RA) into micelles (Fig. 1b). The researchers coated IONPs with OA and encapsulated them within the copolymer micelles. They finally loaded DOX into the magnetic micelles. Folate conjugation enhances targeted delivery to cancer cells with overexpressed folate receptors [41]. The amphiphilic copolymer of FA/DEX-RA forms micelles, providing a hydrophilic outer layer. As FA enhances targeting specificity, OA coats the IONPs and is present in the hydrophobic core of the micelles. This hydrophobic environment facilitates the loading of DOX.

Micelle formation improves solubility and stability of the drug payload [35, 125]. Varshosaz’s group demonstrated that reduced protein binding significantly lowered IC50 of the DOX drug compared to free drug on MCF-7 and MDA-MB-468 cells. They further demonstrated a potential for dual tumor targeting (magnetic field-guided and folate-targeting) using their formulation design [127]. Huang’s group co-assembled poly(γ-glutamic acid-co-distearyl γ-glutamate) with poly(lactic-co-glycolic acid), paclitaxel (PTX), and OA-coated IONPs (Fig. 1c) [53]. Co-assembly with poly(lactic-co-glycolic acid) improves biocompatibility and provides sustained drug release [1]. The researchers utilized adipose-derived stem cells (ADSCs) for selective delivery of nanotherapeutics toward brain tumors. The nanoparticles are designed with a co-assembly of poly(γ-glutamic acid-co-distearyl γ-glutamate) and poly(lactic-co-glycolic acid), suggesting a hydrophilic outer layer. The nano formulation was prepared using an oil-in-water emulsion technique, incorporating OA coated IONPs. Phosphate buffer is used to create an aqueous environment during the emulsion process. OA coats the IONPs, providing a hydrophobic environment which allows for the loading of paclitaxel (PTX) drugs [117]. PTX is co-loaded into the hydrophobic core, and the system is designed for combined hyperthermia and chemotherapy, which can be activated by an external high-frequency magnetic field (HFMF). Huang’s group confirmed a successful cellular transport and cytotoxic action of therapeutics against tumor cells in vivo. They demonstrated a fourfold increase in therapeutic index on brain astrocytoma-bearing mice compared to conventional chemotherapy. The group further enabled combined hyperthermia/chemotherapy activated by an external high-frequency magnetic field (HFMF) which led to a significant apoptosis of glioma cells through HFMF-mediated hyperthermia and thermally triggered release of the PTX drug.

#### Polyethylene glycol (PEG) coating

**Fig. 2:**
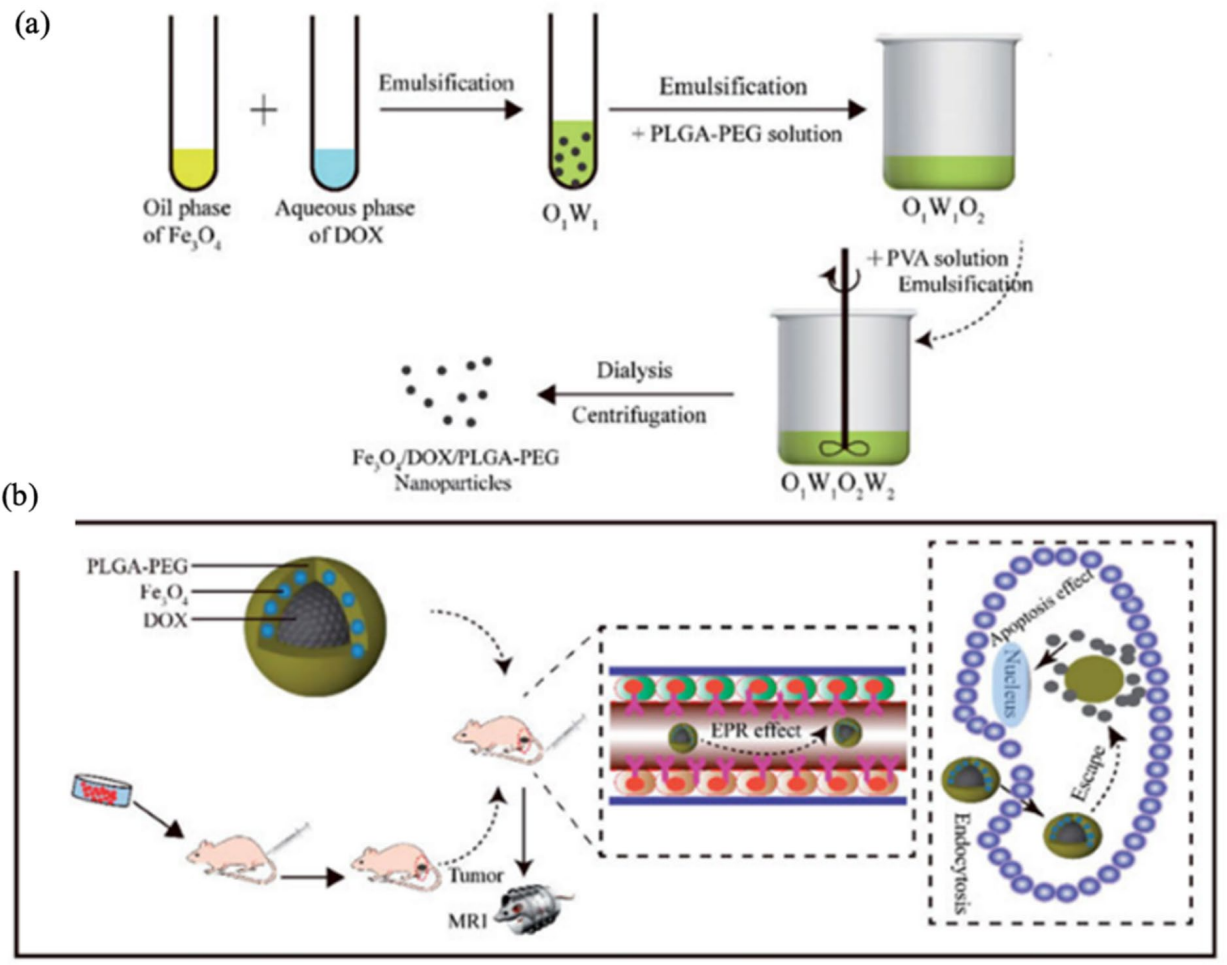
**a** Synthesis of IONP/DOX/PLGA-PEG nanoparticles through the emulsion solvent evaporation technique. **b** A visual representation of the IONP/DOX/PLGA-PEG nanocarrier system, followed by the release of Doxorubicin (Dox) from the nanocarriers for the purpose of eradicating tumor cells. This system serves a dual role in both the early detection and treatment of tumors [73]

Both Dorniani’s group and Liang’s group have made significant contributions to the development of IONPs coated with polyethylene glycol (PEG) for the diagnosis and treatment of cancer [32, 73]. Liang's group used PLGA-PEG to encapsulate IONPs and doxorubicin (DOX) via the emulsion-solvent evaporation method (Fig. 2). PEG chains are water-soluble and provide a hydrophilic surface to the nano conjugate, making it more biocompatible and improving its circulation time in the bloodstream. PEG also helps in reducing nonspecific interactions with biological components [73]. PLGA is a biodegradable and hydrophobic polymer that forms the core of the nano conjugate, providing a hydrophobic environment for the encapsulation of the hydrophobic drug (DOX) and IONPs. This step involved the conjugation of carboxyl (COOH) groups on PLGA to amine (NH2) groups on PEG, forming a covalent bond. This connection ensures the integration of the hydrophilic PEG chains with the hydrophobic PLGA polymer, creating an amphiphilic copolymer [111]. The IONPs are hydrophobic and are encapsulated within the hydrophobic core of PLGA-PEG. This encapsulation is crucial for preventing aggregation of the IONPs and providing stability to the nanostructure. The hydrophobic anticancer drug DOX was also encapsulated within the hydrophobic core of PLGA-PEG. This encapsulation protects the drug from premature release, allowing controlled drug release at the target site. Liang’s group observed a cytocompatibility effect in NIH 3 T3 fibroblast cell culture while using their nanocomposite. At a concentration of less than 1 mg/mL, the nanocomposite hardly had any adverse effects on RBC aggregation, morphology, RBC lysis, or blood coagulation. The researchers additionally observed an enhanced T2 contrast magnetic resonance with a relatively high activity of released DOX within 120 h on human breast cancer MCF-7 cells. Dorniani’s group coated IONPs with PEG to form pegylated IONPs and then loaded the drug 6-mercaptopurine (MP) into the FPEGMP-2 nanocomposite, with an estimated drug loading of about 5.6%. Additionally, Dorniani's group designed IONPs with PEG alone as compared to Liang’s group that used PLGA-PEG. Using PEG alone for surface modification is a simpler approach. PEGylation involves the attachment of PEG chains to the nanoparticles’ surface, creating a hydrophilic shell. This can improve the stability and biocompatibility of the nanoparticles. PEGylation increases the hydrophilicity of the nanoparticles, making them highly dispersible in aqueous solutions [67]. This is important for drug delivery, as the particles need to be well-dispersed to effectively transport drugs. Unfortunately, such conjugates may have limited drug-loading capacity, as PEG primarily serves to improve nanoparticle stability and reduce nonspecific interactions rather than acting as a drug carrier [34]. PEG alone does not provide a controlled release mechanism for the drug [6, 7].

Dorniani’s group noted a controlled release behavior of MP from FPEGMP-2 nanocomposite into phosphate-buffered solution with about 60% and 97% release at pH 7.4 and 4.8, within about 92 and 4.8 h respectively. FPEGMP-2 demonstrated slightly higher anticancer activity on leukemic WEHI-3B cell lines than FPEGMP-0.5 nanocomposite in a dose-dependent manner.

### Silica coating

**Fig. 3:**
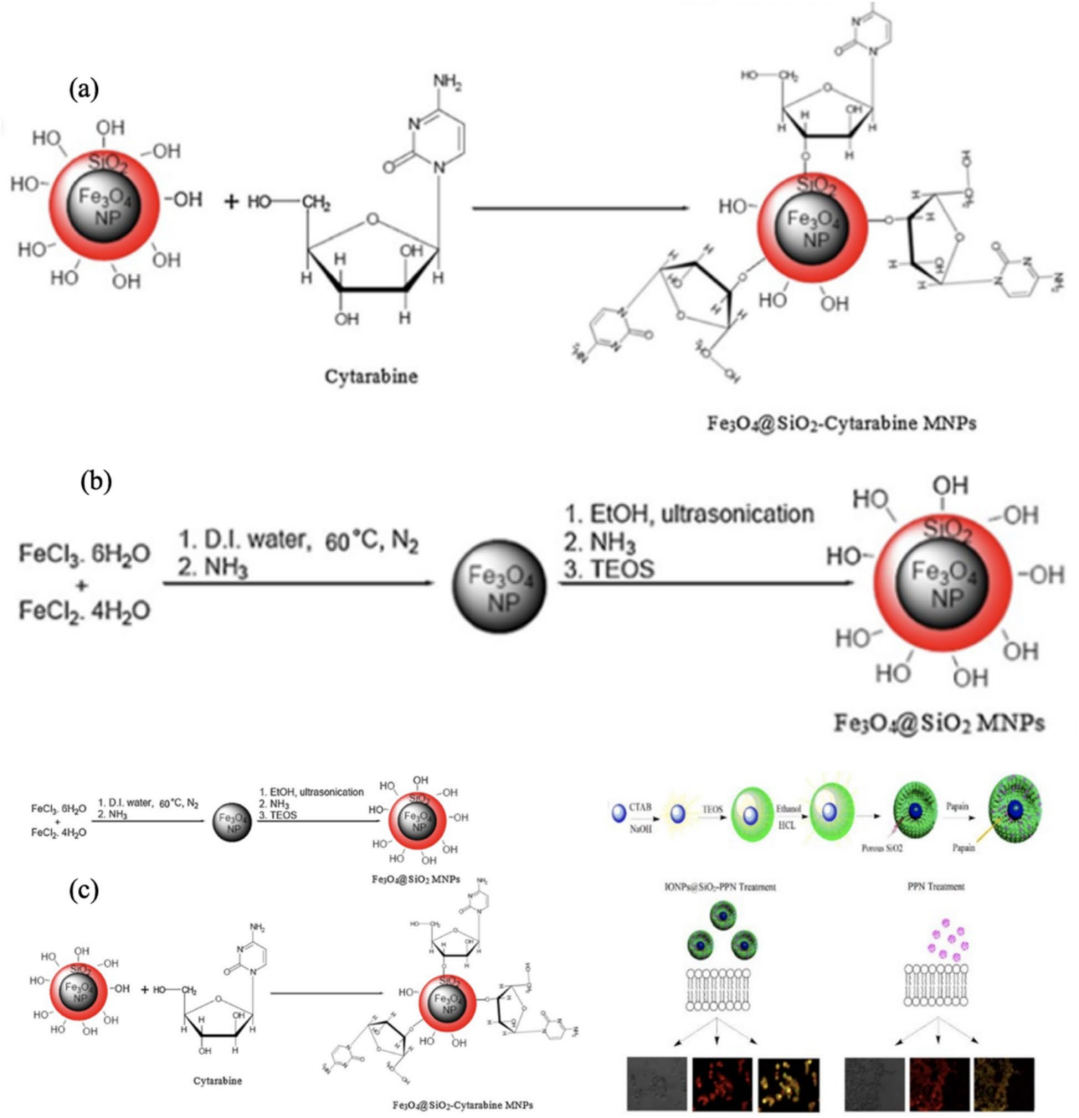
**a** Proposed arrangement of cytarabine bonding on the surface of IONP@SiO2 magnetic nanoparticles [109]. **b** The creation of IONPs@SiO2-PPN. **c** Examinations conducted in vitro [91]

Both Shahabadi’s and Nasiri’s groups have contributed to the design of silica-coated IONPs (IONP-SiO_2_) for potential cancer treatment applications [91, 109]. Shahabadi’s group bound cytarabine onto the surface of IONP-SiO_2_ through the hydroxyl groups (Fig. 3a). Silica is considered to possess a hydrophilic characteristic which contributes to the stability of the IONPs in aqueous solutions, thus preventing their aggregation. The IONPs surface was modified with hydroxyl (-OH) functional groups to control their interactions. This modification enhances the hydrophilic properties and allows for further functionalization [87, 113, 132]. Cytarabine, being a hydrophilic drug, interacts with the hydrophilic regions on the surface of the silica coating with the modified functional groups. This interaction facilitates the encapsulation of the drug onto the IONPs. The IONPs themselves possess a hydrophobic core surrounded by a hydrophilic shell (silica) [14]. This core–shell structure influences the drug-loading capacity and release kinetics. The hydrophobic and hydrophilic interactions between the IONPs and the drug influence the release of the drug. The surface modification was designed to enhance the targeting of specific HL60 cells. The hydrophilic nature of the silica coating additionally affects the interaction of the IONPs with the cell membrane which is crucial for cellular uptake. Shahabadi’s group exhibited a strong cytotoxic effect on HL60 cancer cell lines using MTT colorimetric assay, and the DNA binding studies suggested a groove binding mode, indicating potential for targeted therapy.

As compared to Shahabadi’s group, Nasiri’s group used porous silica to load papain onto IONP- SiO_2_ through a solvent evaporation method (Fig. 3b). The presence of pores in the silica coating increases the surface area available for drug loading. The porous nature of the silica coating allows for a higher drug-loading capacity whereby the size and structure of the pores influence the rate of drug release. Papain, being a protein enzyme, interacts with the hydrophobic regions within the porous silica. This interaction facilitates the encapsulation of papain onto the IONPs. The hydrophobic environment within the pores protects the encapsulated papain from degradation and denaturation, enhancing its stability during transportation and delivery. The porous structure additionally influences the release kinetics of papain. The drug gets released gradually from the pores, providing a controlled and sustained release profile. The hydrophilic nature of the silica coating and the porous structure influence the interaction of IONPs with the cell membrane hence facilitating cellular IONP uptake. Nasiri’s group presented a physiologically compatible and stable drug delivery vehicle, showing reduced IC50 values and enhanced cytotoxic effects compared to native papain onto the HeLa cancer cells. The in vivo acute toxicity assessment revealed no observable clinical indications of discomfort or distress, nor any weight loss 1 week following the intravenous administration of IONPs@SiO2 (10 mg/kg) in 8-week-old Balb/C mice.

### Dextran coating

**Fig. 4:**
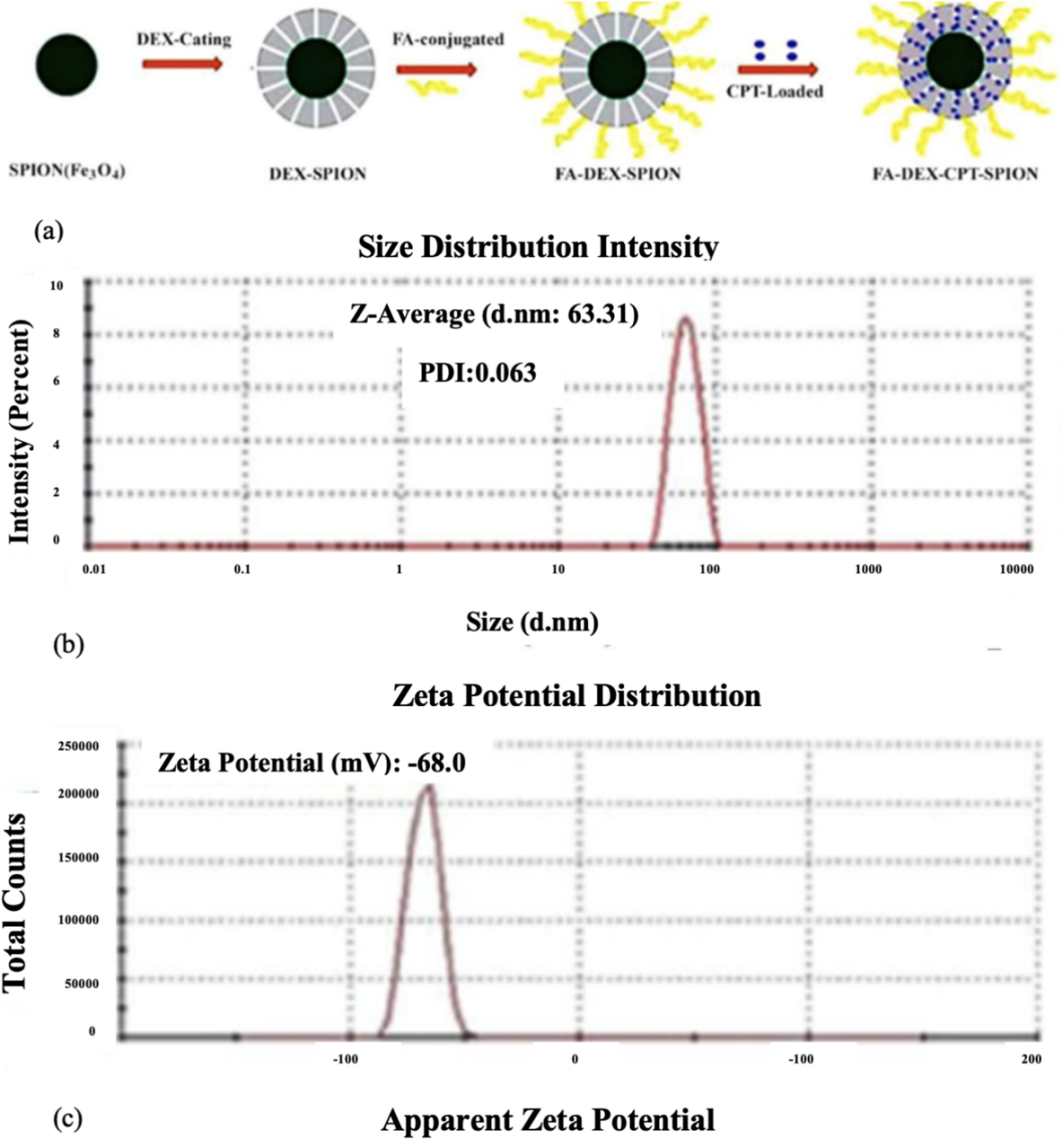
**a** Diagram depicting the preparation process of the FA-DEX-CPT-IONP nanocomposite is presented, showcasing. **b** The Z-average or diameter size andpolydispersity. **c** The charge of the FA-DEX-CPT-IONP nanocomposite [5]

Both Predoi and Al-Musawi’s groups engineered dextran-coated IONPs (DEX-IONPs) for cancer treatment, but with specific differences in the drugs used and the synthesis reagents [5, 9]. Predoi’s group coated 5-Fluorouracil (5-FU) onto the surface of IONPs through the hydroxyl groups provided by dextran. Dextran is a polysaccharide known for its hydrophilic nature. The hydrophilic property of dextran contributes to the IONP stability in aqueous solutions, which further prevents the IONP aggregation. Dextran contains hydroxyl (-OH) groups on its surface, contributing to its hydrophilic nature. 5-FU, being a hydrophilic drug, interacts with hydrophobic regions within the dextran coating. This interaction facilitates the encapsulation of 5-FU onto the IONPs. The IONPs contain a hydrophobic iron oxide core surrounded by a hydrophilic dextran shell. This core–shell structure influences the drug-loading capacity and release kinetics. The hydrophobic and hydrophilic interactions between the IONPs and 5-FU influence the release of the drug. Predoi’s group provided a first-ever study demonstrating the downregulation of Mini-Chromosome Maintenance 2 (MCM-2) expression in Caco-2 cells exposed to DEX-IONPs and conjugated with 5-FU. The group additionally identified that the nano formulation with the concentration ratio (IONP: 5-FU) of 1.5:1 had the highest anti-tumor efficiency as compared to 0.5:1 and 1:1 ratios. In addition to DEX-IONPs, Al-Musawi’s group added folate (FA) onto their design with a focus on targeted delivery and examining release kinetics and anti-cancer efficacy (Fig. 4). Folate is hydrophilic and contains a carboxyl group that can participate in hydrogen bonding. Folate is often used as a ligand to target cancer cells because many cancer cells, including prostate cancer cells, overexpress folate receptors on their surface. The inclusion of folate on the surface of IONPs facilitates active targeting, as the nanoparticles specifically bind to folate receptors on the prostate cancer cells, enhancing uptake. Camptothecin (CPT), being a hydrophobic drug, interacts with hydrophobic regions within the dextran coating. This interaction facilitates the encapsulation of CPT onto the IONPs. The drug release gets controlled, allowing for a sustained and targeted delivery of CPT to the prostate cancer cells. The dextran and folate coating provides stability to CPT during circulation in the bloodstream, protecting it from degradation and enhancing its bioavailability. Folate receptors on the surface of prostate cancer cells facilitate the internalization of the IONPs via receptor-mediated endocytosis, which enhances the efficiency of drug delivery to cancer cells.

Al-Musawi’s group highlighted a pH-dependent drug release for the FA-DEX-IONPs and CPT complexes with a higher CPT release at the pH of 5.4 than the release at the pH of 7.4. The complexes additionally showed that CPT–FA-DEX-IONPs were superior in anti-cancer treatment as compared to CPT drug alone.

## Antibody therapy

**Fig. 5:**
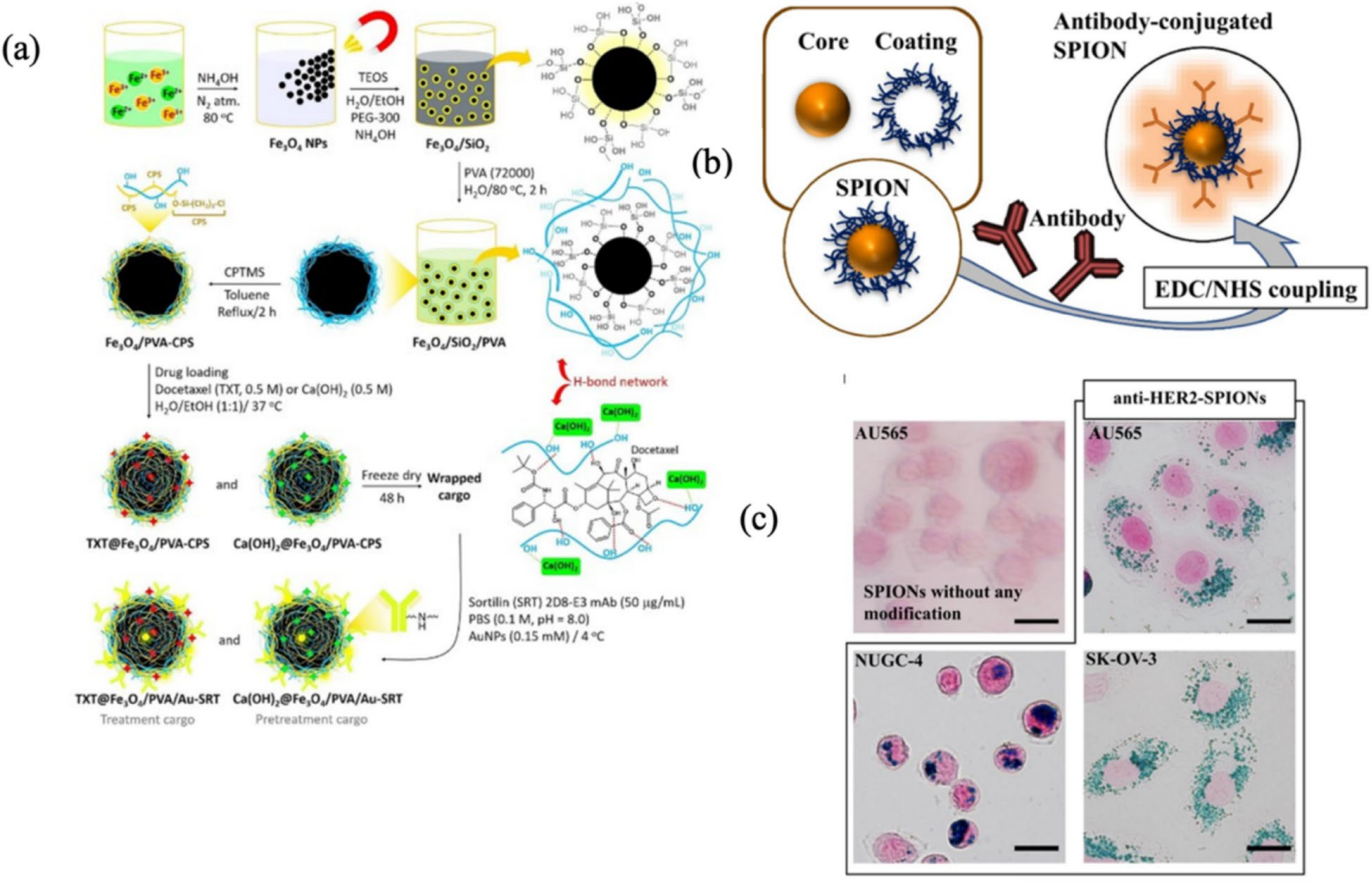
**a** Schematic depiction of the preparation process for TXT@IONP/PVA/Au-SORT nanotherapeutics [121]. **b** The coupling of IONPs with antibodies was accomplished through amine bonds formed between the surface coating of IONPs and the antibody, utilizing the EDC/NHS reaction. **c** The internal delivery of anti-HER2 antibody-conjugated IONPs (with a carboxydextran coating) is illustrated, showing the presence of blue anti-HER2 conjugated IONPs in cancer cells with HER2 overexpression (AU565, NUGC-4, SK-OV-3.[61]

The groundbreaking work conducted by Taheri-Ledari’s group and Kagawa’s group has significantly advanced the field of cancer therapy through the innovative design of IONPs functionalized with antibodies [60, 121]. Taheri-Ledari’s group introduced a unique strategy involving the pretreatment of ovarian cancerous cells with calcium hydroxide followed by treatment with Taxotere (TXT) (Fig. 5).

They designed IONPs functionalized with sortilin antibodies for targeted delivery to caov-4 ovarian cancer cells. The IONPs were hydrophilically stabilized with a silica network, PVA strands and Human IgG1 sortilin antibody (SORT). The incorporation of gold nanoparticles in PVA introduced a hydrophobic element and the plasmonic heating process, which controlled the TXT release. Their system demonstrated high growth inhibition potency and selective cell attachment. A 21-day treatment program, including pretreatment and subsequent therapy, effectively inhibited tumor growth, suggesting a promising strategy for aged tumor tissues.

Kagawa’s group focused on hyperthermia for cancer therapy using IONPs conjugated with an anti-HER2 antibody (trastuzumab) (Fig. 5). The IONPs provided a hydrophobic core onto which carboxydextran was coated. The carboxydextran coated IONPs introduced hydrophilic properties onto which EDC/NHS coupling was used for binding anti-HER2 antibody (trastuzumab) to the IONPs. This novel immuno-magnetic hyperthermia technology selectively targeted breast cancer cells (AU565), human gastric cancer cells (NUGC-4) and human ovarian cancer cells (SK-OV-3), inducing apoptosis while sparing normal cells (Fig. 5c).

## Gene therapy

In the realm of cancer therapy, various research groups have pioneered innovative strategies utilizing IONPs for targeted delivery of therapeutic genes, aiming to enhance the precision and efficacy of treatments. Satake’s group, Yang’s group, and Chung’s group have each devised distinct approaches harnessing the unique properties of IONPs, demonstrating significant progress in the field of gene delivery for cancer therapy [27, 108, 140]. Satake’s group developed a therapeutic concept that combined MXD3 siRNA, anti-CD22 antibodies, and IONPs for targeted therapy against precursor B-cell acute lymphoblastic leukemia (preB ALL). The developed therapy utilizes hydrophilic siRNA and antibodies for targeted gene silencing, combined with the hydrophobic core of IONPs for stability and efficient delivery to preB ALL cells. The IONP core provides a hydrophobic environment that allows them to interact physically with the hydrophilic siRNA and aCD22 Ab molecules to form stable nanocomplexes. The hydrophilic siRNA and antibodies interact with water molecules, while the hydrophobic core of IONPs facilitates stable complex formation, ensuring targeted delivery and gene silencing. Electrostatic interactions between the negatively charged siRNA molecules and the amine modified IONPs facilitate adsorption of siRNA on the nanoparticle surface. Antibodies are physically adsorbed onto the NP surface, contributing to the overall stability of the nanocomplex. siRNA carries the genetic information and is designed to target and silence the MXD3 gene in preB ALL, whereas aCD22 Ab facilitate interactions with water molecules. The MXD3 siRNA effectively entered leukemia cells, leading to the knockdown of MXD3 gene expression. The cytotoxic effects of MXD3 siRNA-aCD22 Ab-IONP complexes were significantly enhanced when combined with chemotherapy drugs (vincristine and DOX). The complexes exhibited potential therapeutic effects on preB ALL cells while sparing normal CD34-positive hematopoietic stem cells and non-B cells.

**Fig. 6:**
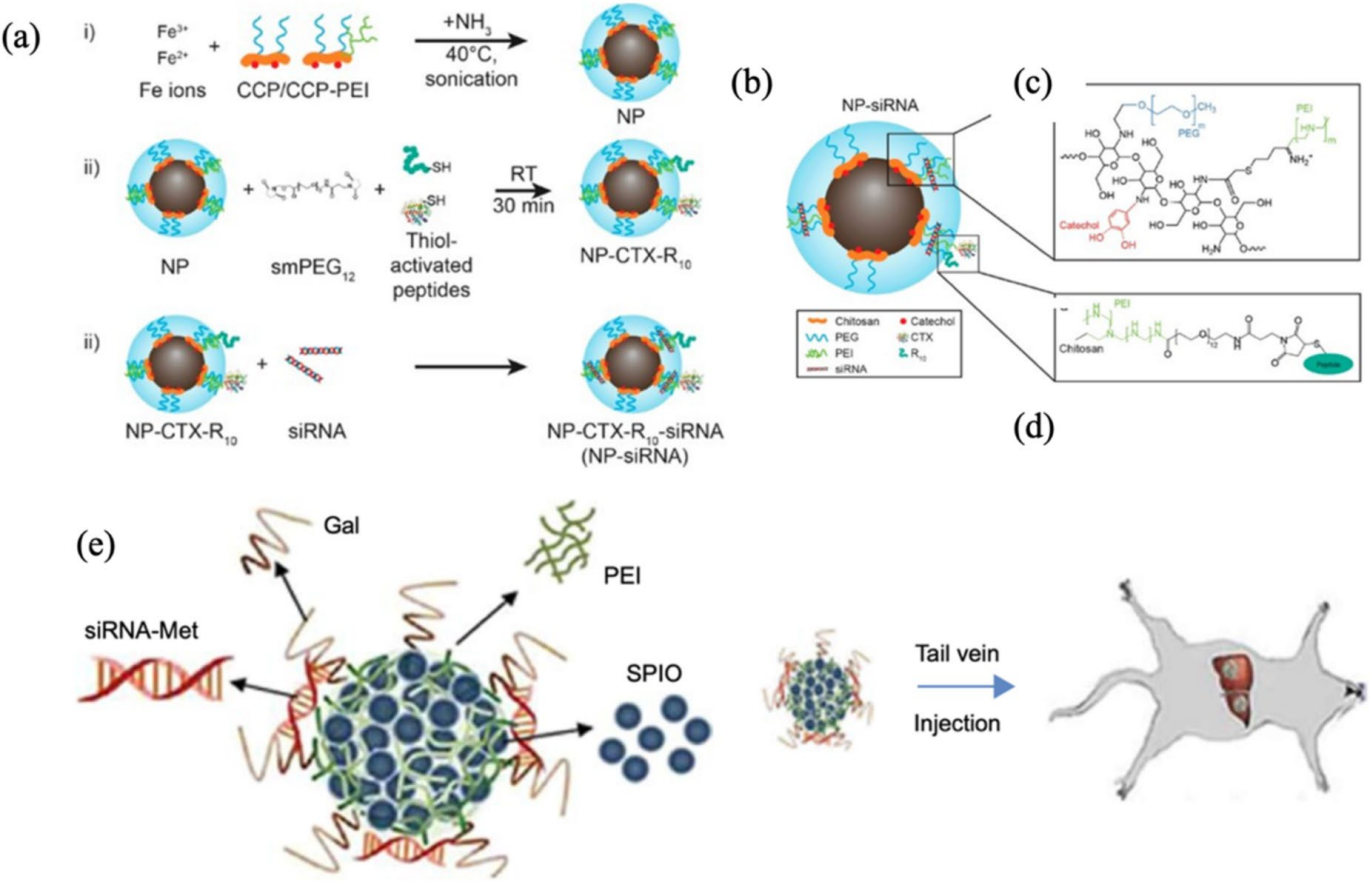
Schematic representation outlining the preparation process of NP-CTX-R10. **a** The synthesis procedure for NP and NP-siRNA complex involves mixing ferric and ferrous ions with catechol-chitosan-PEG(-PEI) (CCP/CCP-PEI) polymers, followed by sonication at 40 °C and continuous ammonia titration to produce the base NPs. Thiol-activated CTX and R10 peptides are then conjugated onto the NP surface using succinimidyl-maleimide-PEG linker. The resulting peptide-modified NPs are complexed with siRNA through electrostatic interaction. **b** A schematic diagram illustrates the design of the siRNA delivery vector. **c** The chemical structure of CCP-PEI copolymer is depicted. **d** The detailed structure of the linkage between CCP-PEI and the conjugated peptide [27]. **e** A schematic illustration demonstrating the encapsulation of Gal-PEI-IONP with siRNA and its injection into mice [140]

Yang’s group demonstrated a study involving the hydrophilic nature of Galactose (Gal), PEI, and siRNA, with the hydrophobic contribution of IONPs for the effective binding, protection, and delivery of siRNA to liver cancer cells (Fig. 6b). Polyethylenimine (PEI) is hydrophilic, aiding in the formulation of stable nanoparticles. Gal is hydrophilic and serves as a targeting ligand for specific receptors on hepatocellular carcinoma (HCC) cells. The hydrophobic core of IONPs serves as a carrier for the siRNA, Gal, and PEI. Electrostatic adsorption of PEI onto the surface of IONP-COOH and subsequent attachment of Gal-PEG-NH2 contribute to the synthesis of Gal-PEI-IONPs. The nanoparticles complex with siRNA, formed stable Gal-PEI-IONP/siRNA complexes. Gal-PEI-IONP shield siRNA from serum degradation, prolonging the half-life of siRNA in the system. Gal-PEI-IONP encapsulation protects siRNA from nuclease degradation, extending its half-life in the system. Gal-PEI-IONPs, with an IONP core modified by galactose and polyethylenimine, facilitated targeted delivery of therapeutic siRNA to liver cancer cells. The nanoparticle complex efficiently entered Hepa1–6 cells of the tumor-bearing mice and reduced tumor volume and liver/body weight ratio, hence demonstrating an effective therapeutic effect.

Chung’s group developed IONPs functionalized with peptides (NP-CTX-R10) for targeted siRNA delivery to silence the MGMT gene in glioma cells (Fig. 6a). The hydrophobic IONP core was coated with catechol-chitosan-PEG-PEI copolymer which provided hydrophilic regions due to chitosan and PEG. The catechol-chitosan-PEG-PEI copolymer onto the IONPs was functionalized with functional peptides (CTX and R10) that contain hydrophobic and hydrophilic regions. Peptide functionalization with chlorotoxin (CTX) and polyarginine (R10) enhances the transfection capability of siRNA comparable to commercially available Lipofectamine. Polyarginine R10 facilitates endosomal escape, allowing sufficient presence of siRNA in the cytosol while avoiding endosomal degradation. Hydrophilic siRNA was then attached to the functional peptides, facilitating interactions with water-soluble components in the cellular environment. Electrostatic interactions between the hydrophilic siRNA and the hydrophilic regions of peptides contribute to the formation of stable nanocomplexes. The catechol group improves stability by interacting with iron, enhancing the interaction between the polymer coating and the IONP core. Functional peptides, CTX and R10, contribute to effective siRNA delivery and gene silencing in glioma cells. The NP-siRNA complex achieved up to 90% gene silencing, demonstrating the effectiveness of the siRNA delivery system. Glioma cells transfected with NP-siRNA targeting MGMT showed elevated sensitivity to the alkylating drug Temozolomide (TMZ).

## Imaging and diagnostics

**Fig. 7:**
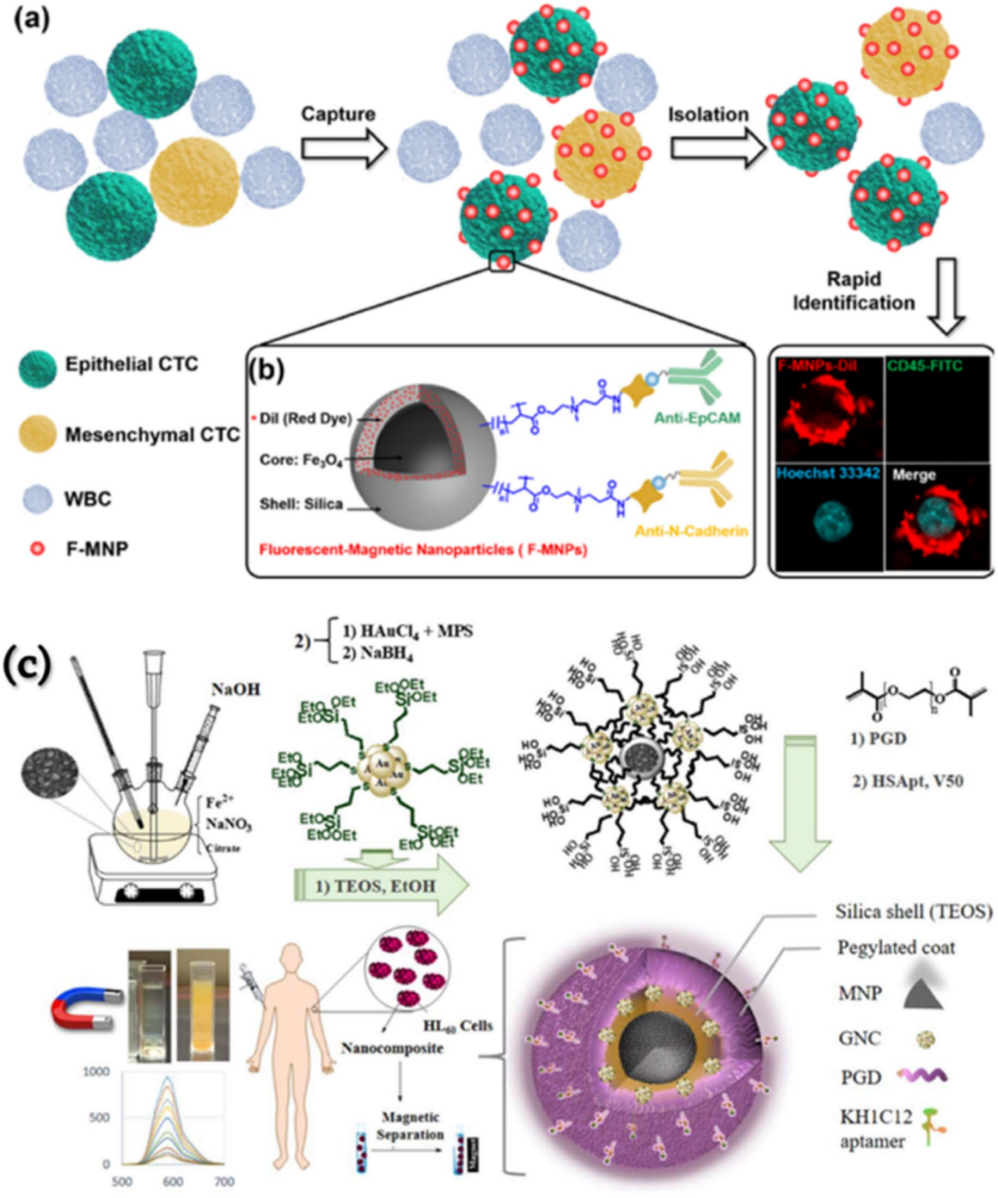
Visual representations demonstrating (**a**) the swift isolation and identification of diverse circulating tumor cells (CTCs) using fluorescent-magnetic nanoparticles (F-MNPs), (**b**) the assembly and chemical modification of F-IONPs [131]. (**c**) A diagram outlining the synthesis of the IONP/GNCs/Aptamer nanoprobe [49]

Three research groups have made significant contributions to the field of cancer diagnostics and theranostics by employing innovative approaches involving hydrophilic modifications, antibody targeting, shape engineering, and dual-imaging capabilities [19, 49, 128, 131]. Each study addresses different aspects of CTC isolation, detection, and imaging, collectively contributing to advancing biomedical applications and cancer research. The combination of fluorescent gold nanoclusters (GNCs) and IONPs allows for dual-imaging capabilities, enabling both fluorescence imaging and T2-based contrast-enhanced MRI. This concept was verified by Haghighi’s group as they designed an aptamer nanoprobe which facilitates targeted diagnosis and dual-imaging of highly malignant HL-60 cancer cells (Fig. 7). The hydrophobic core of IONPs was shielded by the hydrophilic silica layer to avoid nonspecific interactions and make them stable in aqueous environments. The hydrophobic mercaptopropyl group provided a hydrophobic interaction interface for the GNCs and contributed to the stability of the thiol-modified GNCs. The GNCs enhance the magnetic properties for T2-based MRI, and their presence enables fluorescent microscopy, providing a comprehensive imaging approach. The thiol modification introduced the hydrophobicity segment, facilitating the conjugation through thiol-en click reaction with the double bond groups of polyethylene glycol) dimethacrylate (PGD). The Thiol-Modified Aptamer sequence thus provides hydrophilic regions that interact specifically with target HL-60 cells. The use of (γ-Mercaptopropyl) trimethoxysilane (MPS) and poly(ethylene glycol) dimethacrylate (PGD) contributes to the stability, excellent colloidal and photo stability, and cytocompatibility of the IONP/GNCs nanoprobe. The trimethoxysilane group is hydrophilic and interacts with polar molecules and surfaces, providing stability in aqueous environments. This helps in the functionalization and stabilization of gold nanoclusters (GNCs). The IONP/GNCs/KH1 C12 aptamer nanoprobe facilitated targeted diagnosis and dual-imaging of highly malignant HL-60 cancer cells. The nanoprobe demonstrated high sensitivity, detecting as few as 10 cells in a range of cancer cells, with the fluorescence intensity increasing with cell numbers, thus enabling quantification and sensitivity assessment.

Wang’s group developed a rapid method that ensures high efficiency in isolating heterogeneous circulating tumor cells (CTCs) from patient blood samples (Fig. 7a, b) [128, 131]. The carboxyl groups were attached on the IONPs surface to provide a hydrophilic segment suitable for further functionalization. Fluorescent dyes (DiI) were then added to contribute to water solubility and stability with their hydrophilic nature within the biological fluids. This created fluorescent IONPs (F-IONPs) which were further surface modified with dual antibodies and the overall design enhanced hydrophilicity, making the F-IONPs suitable for use in blood samples. The hydrophilic dual-antibody interface on fluorescent-magnetic nanoparticles (F-MNPs) comprised of both EpCAM and N-cadherin, allowing for the capture of both epithelial and mesenchymal CTCs. Dual-antibody interface targeting EpCAM and N-cadherin on F-MNPs enables the isolation of CTCs, hence addressing the challenges posed by the rarity and heterogeneity of CTCs. Carboxybetaine groups are highly hydrophilic, imparting excellent antifouling properties and preventing nonspecific adhesion to nontargeted cells. The use of poly(carboxybetaine methacrylate) (pCBMA) brushes reduces nonspecific cell adhesion, enhancing the efficiency and purity of CTC isolation. F-IONPs provided a rapid identification strategy for heterogeneous CTCs within 1 h after isolation, streamlining the diagnostic process. This functionality was demonstrated not only in artificial samples but also in real patient blood samples, emphasizing the translational potential for clinical use.

Chang’s group carried out a study that focused on the impact of different morphologies (spherelike vs. rodlike) of F-IONPs on the isolation and detection of CTCs [19]. Different IONP morphologies (spherelike vs. rodlike) were stabilized with Polyacrylic acid (PAA) to enhance their water solubility characteristics. The mesoporous silica structure was then attached to provide both hydrophilic and hydrophobic regions. Antibody of epithelial cell adhesion molecule (EpCAM) was then attached onto these different morphologies to provide a hydrophilic segament. Conjugation with the EpCAM antibody enhances the efficiency of CTC enrichment and fluorescent-based detection, contributing to the sensitivity and specificity of the method. Antibodies generally have hydrophilic regions, enabling specific interactions with target cells like Michigan Cancer Foundation-7 (MCF-7) breast cancer cells and CTCs. The silica shell and the overall design provide hydrophilicity, ensuring colloidal stability and compatibility in biological environments. Both differently shaped IONP morphologies demonstrated good sensitivity for the isolation and fluorescence detection of CTCs, with the aid of the EpCAM antibody. However, rodlike IONP shapes outperformed spherelike counterparts in terms of immunomagnetic isolation and detection, showcasing the importance of shape engineering for optimal performance.

## Photothermal therapy

**Fig. 8:**
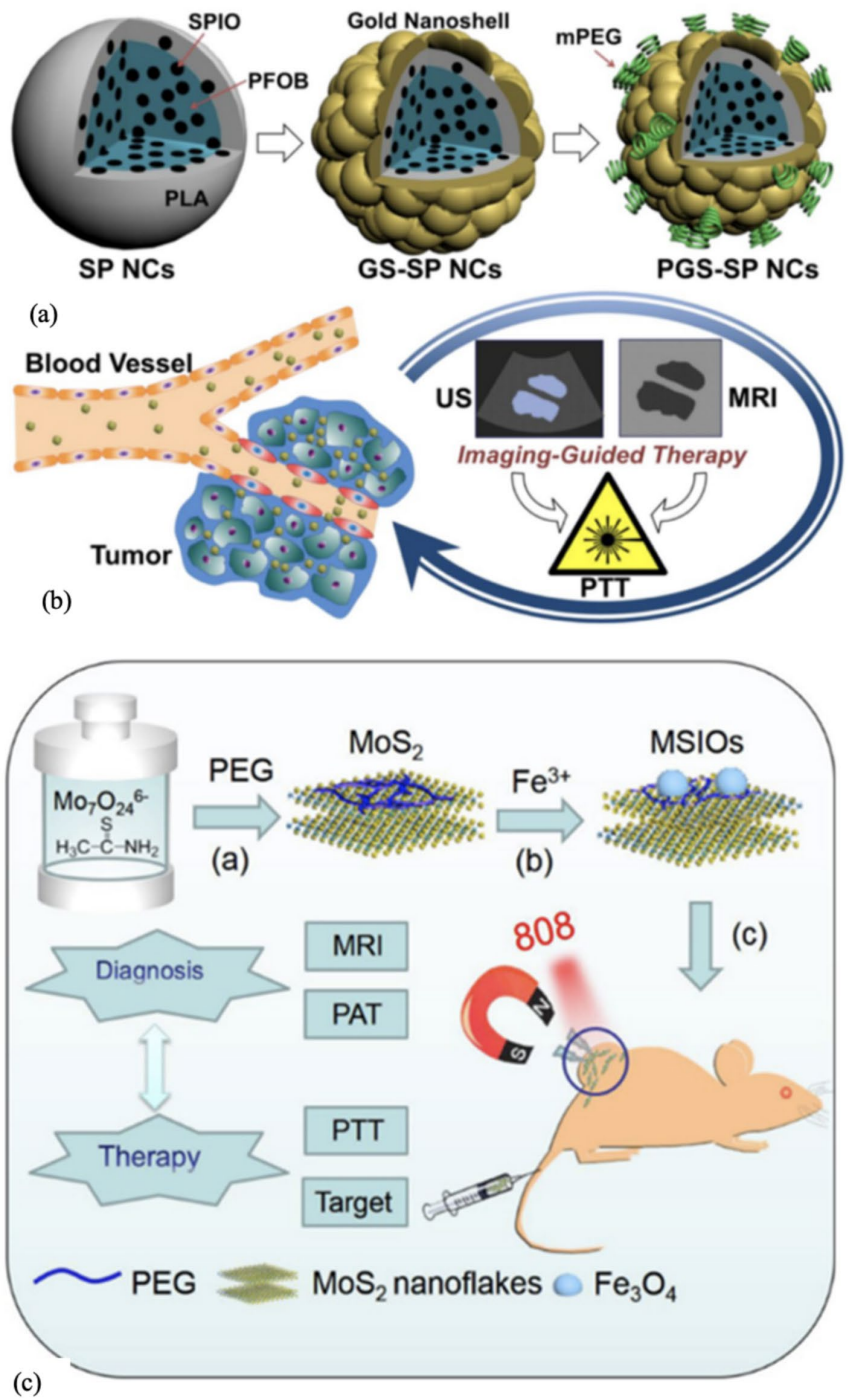
**a** Schematic representation of the manufacturing process of PGS-SP NCs; **b** The bimodal US/MRI-guided tumor photothermal therapy (PTT) process employing the nanotheranostic agen [63]. **c** Diagram depicting the synthesis pathway and theranostic steps of MoS2/IONP composite for dual-modal MR and PAT imaging-guided magnetic targeting for photothermal ablation of cance [141].

Both Yu's and Ke's groups have made significant contributions to the field of cancer theranostics by designing multifunctional composites for effective photothermal ablation, accurate diagnosis, and therapy guidance [63, 141]. While both approaches involve the use of IONPs and include PEGylation for enhanced stability, there are notable differences in their methodologies and components. Yu’s group designed an IONP composite that integrates photothermal therapy with magnetic targeting and dual-modal imaging, offering the potential for effective cancer treatment with minimal side effects (Fig. 8). The two-step hydrothermal method used in the synthesis process involved hydrophobic interactions between the IONPs and the Molybdenum disulfide (MoS2) nanoflakes. PEG was used to functionalize the MoS2/IONP composite, hence contributing hydrophilic properties. PEG enhances the stability of MoS2/IONP in bio-fluids, promoting their biocompatibility and preventing aggregation. The IONPs serve as the target moiety directed by an external magnetic field to the tumor site and contribute to the magnetic properties of the entire composite for magnetically targeted photothermal ablation [100]. MoS2 converts near-infrared (NIR) light into heat, enabling photothermal therapy. MoS2/IONP act as a dual-modal probe for T2-weighted magnetic resonance (MR) and photoacoustic tomography (PAT) imaging which enables comprehensive imaging for diagnostic information and monitoring disease progression. MoS2/IONP composite enabled selective monitoring and destruction of Hela and HepG2 cancer cells through photothermal ablation.

Ke’s group designed a multifunctional nanocapsules model that offered a theranostic approach for effective photothermal ablation, combining bimodal imaging for accurate diagnosis and therapy guidance (Fig. 8c). The construction involved encapsulating IONPs into Poly lactic acid (PLA) nanocapsules, forming a gold nanoshell on the surface, and PEGylation through Au–S linkage. IONPs known for their hydrophobic nature, were encapsulated in the nanocapsules. IONPs contribute to the magnetic resonance imaging (MRI) capability of the nanotheranostic agent. PEGylation of gold nanoshells and PLA nanocapsules adds hydrophilic properties. PLA is a hydrophobic polymer forming the nanocapsule matrix. Perfluoropctylbromide (PFOB) is loaded into the nanocapsules to provide hydrophobic properties. PFOB additionally provides ultrasound (US) contrast enhancement both in vitro and in vivo. The hydrophilic PEG enhances stability, while hydrophobic components such as PFOB, SPIOs, and PLA contribute to imaging and therapeutic properties in a multifunctional nanotheranostic agent. A successful photothermal ablation was achieved in human tumor xenograft models non-invasively while utilizing real-time US and high-resolution MRI imaging for precise treatment.

## Properties of IONPs

IONPs have garnered significant attention in various fields, including medicine, due to their unique and tunable properties. The success of IONPs in applications such as drug delivery, imaging, and hyperthermia therapy relies on their distinctive physical, magnetic, and chemical characteristics. This section explores these fundamental properties that make IONPs versatile tools in the realm of nanotechnology and biomedicine.

## Physical properties IONPs

IONPs exhibit a range of physical properties that make them particularly useful in various applications, from medicine to electronics. IONPs typically have diameters ranging from a few nanometers to around 100 nm. Their small size provides a high surface area-to-volume ratio, which is advantageous for catalysis, adsorption, and drug delivery [4]. One of the most notable properties of IONPs is their magnetic behavior. Depending on the type of iron oxide and its crystal structure, these nanoparticles can be ferromagnetic, ferrimagnetic, or superparamagnetic. This property is the basis for applications such as magnetic hyperthermia, targeted drug delivery, and magnetic resonance imaging (MRI) contrast agents [7, 56, 68, 74]. IONPs can exist in various crystalline forms, including magnetite (Fe_3_O_4_), maghemite (γ-Fe_2_O_3_), and hematite (α-Fe_2_O_3_). The crystalline structure influences their magnetic properties, stability, and reactivity [72]. The surface of IONPs can be functionalized with various molecules, such as polymers, biomolecules, and ligands. This functionalization affects their stability in solution, biocompatibility, and interactions with other materials or biological systems [98, 149]. IONPs can exhibit interesting optical properties, especially when they are engineered to have specific sizes and shapes. Their absorption and scattering of light can be used in photothermal therapies and imaging applications [2, 18]. When IONPs are below a certain critical size, they exhibit superparamagnetism. This means that they do not have a permanent magnetic moment but can become magnetized in the presence of an external magnetic field. Superparamagnetic behavior is useful in medical imaging and magnetic separation techniques [16, 28, 80], 122. Due to their small size and high surface energy, IONPs tend to agglomerate or aggregate in solution. Proper surface coating and functionalization are necessary to achieve stable dispersions and prevent unwanted aggregation [83, 129]. Certain IONPs, particularly those with plasmonic properties, can absorb light energy and convert it into heat. This property is exploited in photothermal therapies, where the heat generated by nanoparticles is used to selectively target and destroy cancer cells [11, 128, 131, 148]. The biocompatibility of IONPs is crucial for their use in medical applications. The surface functionalization and coating determine how well the nanoparticles interact with biological systems and whether they trigger adverse reactions [55, 137, 144]. Some IONPs such as magnetite, exhibit electrical conductivity. This property has implications for their use in electronic and sensing applications [44, 59, 96].

## Magnetic properties IONPs

IONPs exhibit a range of magnetic properties that make them valuable for various applications, particularly in the fields of medicine, electronics, and materials science. The magnetic properties of IONPs are strongly influenced by factors such as particle size, crystal structure, and surface coating. In larger bulk materials, iron oxide compounds like magnetite (Fe_3_O_4_) can exhibit ferromagnetic behavior, where the magnetic moments of individual atoms align in the same direction, leading to a net magnetic moment. However, at the nanoscale, thermal energy becomes more significant, making true ferromagnetism less pronounced due to thermal fluctuations [8, 10, 119]. Many IONPs, including magnetite and maghemite (γ-Fe_2_O_3_), exhibit ferrimagnetism. In this case, the magnetic moments of different atoms within the nanoparticle align in opposite directions, resulting in a net magnetic moment but with reduced overall magnetization compared to ferromagnetic materials [69]. IONPs with sizes below a certain critical value exhibit superparamagnetism. This means that, in the absence of an external magnetic field, the magnetic moments of individual nanoparticles are randomly oriented due to thermal energy. However, when an external magnetic field is applied, the nanoparticles become temporarily magnetized in the direction of the field. Once the field is removed, they lose their magnetization quickly due to thermal fluctuations. Superparamagnetic nanoparticles have a characteristic temperature called the “blocking temperature.” Below this temperature, thermal energy is not sufficient to overcome the energy barrier that prevents the magnetic moments from flipping. Above the blocking temperature, the nanoparticles lose their superparamagnetic behavior [6, 64, 85, 142]. Larger IONPs or those with stronger magnetic interactions may exhibit magnetic hysteresis, a property where the magnetization of the material lags changes with the applied magnetic field. Magnetic hysteresis is a characteristic of ferromagnetic and ferrimagnetic materials [17, 29]. IONPs can exhibit magnetic anisotropy, meaning that their magnetic properties are directionally dependent. This anisotropy can arise from the crystal structure and particle shape and affects the stability of the nanoparticles’ magnetic alignment [16, 38, 40, 126]. The magnetic properties of IONPs are crucial for applications like magnetic hyperthermia and targeted drug delivery in cancer treatment. In magnetic hyperthermia, the nanoparticles’ superparamagnetic behavior is exploited to generate heat when exposed to alternating magnetic fields. In targeted drug delivery, the magnetic properties allow for precise localization and guidance of nanoparticles to specific tumor sites [37, 71, 82]. IONPs are used as contrast agents in MRI due to their strong magnetic properties. They alter the local magnetic field, leading to changes in the relaxation times of nearby water protons, which enhances the contrast between different tissues in imaging [28, 65, 90].

## Chemical properties IONPs

IONPs exhibit a range of chemical properties that influence their behavior, reactivity, and applications. These properties are determined by factors such as the type of iron oxide, crystal structure, surface chemistry, and functionalization. IONPs consist of iron (Fe) in various oxidation states, including Fe(II) and Fe(III). The specific oxidation state influences the magnetic, electronic, and redox properties of the nanoparticles [47, 70]. The surface of IONPs can be modified through functionalization with various molecules, such as polymers, ligands, biomolecules, and surfactants. Surface chemistry affects their stability in solution, interactions with other substances, and biocompatibility [149]. Depending on the surface functionalization and the pH of the environment, IONPs can carry positive or negative charges. These surface charges influence their interactions with biological systems, stability in solution, and ability to bind to other molecules [20, 89, 101, 135]. IONPs can undergo redox reactions, where they exchange electrons with other substances. This property is crucial for applications such as catalysis, where the nanoparticles participate in oxidation–reduction reactions [102, 139]. Certain IONPs, such as magnetite (Fe_3_O_4_), exhibit catalytic activity due to their redox properties. They can participate in reactions that involve electron transfer, making them useful in environmental remediation and chemical synthesis [15, 103]. IONPs have a high surface area, which makes them effective adsorbents for various substances, including heavy metals, organic pollutants, and biomolecules. Their adsorption capacity is influenced by surface chemistry and can be tailored for specific applications [92, 99, 112, 114]. The chemical properties of IONPs play a crucial role in determining their biocompatibility. Proper surface functionalization and coatings are necessary to ensure that the nanoparticles do not trigger adverse reactions when introduced into biological systems [55, 137, 144]. IONPs can exhibit pH-sensitive behavior due to changes in their surface charge and structure at different pH levels. This property can be exploited in drug delivery systems that respond to the pH environment of specific tissues [45, 94]. The reactivity of IONPs is influenced by the availability of surface sites for chemical reactions. Depending on their crystal structure and surface modification, nanoparticles can have varying levels of surface reactivity. IONPs can form complexes with various ligands and ions due to their surface chemistry. This property can be utilized for functionalization, sensing applications, and controlled release of molecules. The chemical stability of IONPs can impact their behavior in different environments. Understanding their susceptibility to degradation is important for predicting their behavior over time, particularly in biological systems. Some IONPs exhibit interesting photochemical properties, including the ability to generate reactive oxygen species under certain conditions. These properties are relevant for applications such as photocatalysis and photodynamic therapy.

## Challenges and future perspectives

While IONPs have shown immense promise in various biomedical applications, their successful translation from the laboratory to clinical settings still holds challenges. Addressing these challenges is essential for realizing the full potential of IONPs in fields such as drug delivery, imaging, and therapeutic interventions. In this section, we have explored key challenges associated with IONP usage and outlined future perspectives, emphasizing the potential application of alternating magnetic fields in leukemia treatment.

## Biodistribution of IONPs

Presently, IONPs are of extensive use across diverse fields, particularly in the medical sciences. However, a limited number of studies have investigated the uptake and biodistribution of IONPs [39, 106, 107, 110]. The biological distribution of IONPs is influenced by their size, shape, and surface characteristics, determining factors such as opsonization (serum protein interaction) and particle-cell interaction [25]. Various biodistribution studies indicate the likely localization of IONPs in blood, spleen, liver, and kidneys, with a preference for accumulation in the liver and spleen [97]. Some research highlights the potential use of ultrasmall IONPs as highly effective MRI contrast agents, playing a crucial role in visualizing bio-events such as gene expression and metastasis at cellular and subcellular levels [110]. Biodistribution data, in conjunction with IONP levels, reveal the involvement of both the kidney and liver in IONP elimination. Notably, studies indicate that over 50% of injected IONPs are found in the liver after 6 h, underscoring the role of the reticular endothelial system in IONP clearance and posing a significant challenge in the biomedical application of these particles. The enhanced vascularization and permeability of IONPs contribute to their uptake by the reticular endothelial system and macrophages [43].

## Bioelimination of IONPs

Opsonization stands out as a crucial process in the removal of IONPs from circulation through liver macrophages [84, 97]. In various investigations, the accumulation of IONPs has been observed in the lungs, attributed to their vascularized and monocyte-rich nature [36]. Notably, Chaves et al. [22] observed the presence of IONPs in mouse lungs for a duration of up to 3 months, with no discernible toxicity. The human body typically contains hemoglobin protein, myoglobin, transferrin, and ferritin, constituting 65%, 4%, 0.1%, and 15–30% of IONPs, respectively. The degradation of IONPs is believed to occur similarly to ferritins at the molecular level. This degradation process leads to an elevation in IONP levels in organs, with IONP regulation primarily governed by ferritin and transferrin, which are the two key IONP-protein complexes involved in the storage and transportation of iron ions [105]. Pioneering researchers Nissim and Robson, along with Richter, reported the in vivo biodegradation of IONPs, elucidating the roles of ferritin and transferrin in the biodistribution of degradation products. The mononuclear phagocytic system is responsible for degrading intravenously administered NPs (> 15 nm), generating IONP-protein complexes like hemosiderin and ferritin. Transferrin can also originate from ferritin, being transported to the bone marrow as a precursor for hemoglobin [133] Myoglobin, another essential IONP-protein complex, facilitates oxygen transport in muscles. As aged red blood cells rupture in the spleen, releasing hemoglobin, macrophages metabolize hemoglobin into ferritin, stored in hepatocytes or converted to transferrin for use in red blood cell synthesis [105].

## Concerns in the production of IONPs

The production of IONPs involves several considerations and potential concerns, especially when these nanoparticles are intended for biomedical or environmental applications. Ensuring the safety, reproducibility, and scalability of production methods is crucial. Here are some key concerns associated with the production of IONPs:

### Synthesis methods

The synthesis methods employed in the production of IONPs are a critical aspect that can significantly impact the characteristics, performance, and suitability of these nanoparticles for various applications. Achieving consistent and reproducible synthesis of IONPs is a challenge. Variability in reaction conditions, precursor concentrations, or reaction times can lead to differences in particle size, shape, and properties, affecting the reliability of IONP production [12, 13]. The size of IONPs is a crucial parameter, influencing their magnetic properties, biodistribution, and overall performance. Controlling and maintaining precise size distributions during synthesis can be challenging and is essential for tailoring IONPs to specific applications. Synthesis methods suitable for laboratory-scale production may not be easily scalable for industrial or clinical applications.

Ensuring scalability while maintaining the desired characteristics poses a challenge in producing IONPs on a larger scale [12, 13]. The cost of production is a significant consideration. Some synthesis methods may involve expensive reagents, intricate equipment, or complex procedures, making the overall process economically impractical for large-scale manufacturing. The synthesis process can introduce impurities or contaminants that may affect the biocompatibility and safety of IONPs. Ensuring high purity is crucial, especially when considering biomedical applications where stringent safety standards are essential [52]. Certain synthesis methods may produce by-products or residues that can interfere with the properties of IONPs. Effective purification steps are required to remove unwanted components and ensure the quality of the final nanoparticle product. Some synthesis methods may involve high energy input or elevated temperatures, contributing to increased energy consumption. Developing eco-friendly and energy-efficient synthesis approaches is a consideration for sustainable production. Different synthesis methods can result in variations in the crystal structure of IONPs. The crystal structure influences the magnetic properties, and achieving precise control over this parameter is essential for tailoring IONPs for specific applications [30, 123].

### Surface coating and functionalization

Surface coating and functionalization are critical considerations in the production of IONPs, as they significantly influence the stability, biocompatibility, and functionality of the nanoparticles [118]. The choice of surface coating materials is crucial to ensure biocompatibility and minimize potential adverse reactions when IONPs are used in biological systems. Incompatibility with biological tissues or cells may lead to toxicity concerns. Inadequate surface coating can result in poor stability of IONPs, leading to aggregation or agglomeration. This can impact the uniformity of particle size and distribution, affecting the performance and applications of IONPs. Surface coatings can alter the magnetic properties of IONPs. Achieving a balance between an effective coating for stability and maintaining desired magnetic characteristics is challenging [61].

Functionalization of the IONP surface is often necessary for specific applications, such as targeted drug delivery or imaging. Ensuring the successful attachment of targeting ligands without compromising the overall stability and functionality of the nanoparticles requires careful consideration [62]. The surface coating should allow for easy and controlled modification of IONPs for various applications. Accessibility for functional group attachment or additional modifications is important for tailoring IONPs to specific needs. Ensuring the long-term stability of surface-coated IONPs is critical for storage, transportation, and prolonged use. Factors such as environmental conditions, pH changes, or interactions with biological fluids may impact stability over time. The surface coating may induce immune responses in some instances, leading to clearance by the body’s defence mechanisms. Understanding and minimizing immunogenicity is crucial, especially for applications involving prolonged exposure or repeated administrations [86]. The surface coating must be compatible with the loaded therapeutic agents for drug delivery applications. Ensuring that the coating does not compromise the stability or efficacy of the encapsulated drugs is a concern. The chosen surface coating method should be scalable for mass production while maintaining consistency and quality. Achieving reproducibility on a large scale is crucial for industrial and clinical applications.

### Biocompatibility and toxicity

Biocompatibility and toxicity are paramount considerations in the production of IONPs, especially when these nanoparticles are intended for biomedical applications [144]. The introduction of IONPs into biological systems raises concerns about their potential adverse effects on living organisms. Biocompatibility is crucial to ensuring that IONPs do not cause harm to cells, tissues, or organs when administered for medical purposes. IONPs may trigger immune responses in the body, leading to inflammation or immune reactions. The surface characteristics and coatings of IONPs play a crucial role in minimizing immunogenicity and ensuring compatibility with the immune system [86]. The potential accumulation of IONPs in specific organs, such as the liver or spleen, can be a concern. Understanding the biodistribution and clearance mechanisms is essential to assess the potential long-term impact on organ function. The size and shape of IONPs can impact their interaction with biological structures. Smaller nanoparticles may have different toxicological profiles compared to larger ones, and certain shapes may induce specific biological responses [137]. Inadequate or inappropriate surface coatings may lead to toxicity concerns. The choice of coating materials and their impact on the overall stability, biocompatibility, and cellular interactions must be carefully considered [115]. Over time, IONPs may release iron ions, which can potentially induce toxicity. Controlling the rate of ion release and minimizing the release of toxic ions are critical factors in ensuring the safety of IONP-based products. Understanding how IONPs interact with cells and cellular components is crucial. Excessive cellular uptake or interference with cellular processes may lead to cytotoxicity and impact the overall biocompatibility of IONPs [106, 107].

## Future perspective: how alternating magnetic field (AMF) could be used in Leukemia treatment as a future perspective?

The application of AMF using IONPs holds great promise as a future perspective in leukemia treatment. This innovative approach, often referred to as magnetic hyperthermia therapy, leverages the unique magnetic properties of IONPs to induce localized heating in response to an AMF [26]. IONPs are introduced into the bloodstream and accumulate in leukemia cells. When exposed to an AMF, the IONPs generate heat through magnetic relaxation, leading to selective hyperthermia in leukemia cells. The controlled and localized temperature increase can induce cellular damage and apoptosis specifically in the targeted cancer cells. The localized hyperthermia induced by AMF using IONPs enhances the therapeutic efficacy of leukemia treatment. Elevated temperatures can sensitize cancer cells to conventional therapies, such as chemotherapy or radiation, making them more susceptible to destruction [81]. The selectivity of this approach for leukemia cells, where IONPs are preferentially taken up, helps minimize damage to surrounding healthy tissues. The ability to focus the hyperthermic effects on cancerous cells is crucial for reducing side effects associated with traditional treatments. The heat generated by IONPs in response to the AMF can be precisely controlled, allowing for a tailored and optimized treatment strategy.

This control is important for achieving therapeutic temperatures within the desired range for effective cancer cell eradication. Magnetic hyperthermia therapy using IONPs can be integrated into multimodal treatment approaches [143]. Combining this method with other therapeutic modalities, such as chemotherapy or immunotherapy, may result in synergistic effects, enhancing overall treatment outcomes for leukemia patients [146]. Magnetic hyperthermia therapy is a non-invasive procedure, as the AMF can penetrate tissues without the need for surgical intervention. This characteristic makes it a potentially attractive option for leukemia patients who may benefit from a less invasive treatment approach [24]. The magnetic properties of IONPs also allow for imaging using magnetic resonance imaging (MRI). This enables real-time monitoring of nanoparticle distribution and the effectiveness of hyperthermia therapy, providing valuable feedback for treatment optimization.

## Conclusion

The diverse landscape of IONPs in the realm of leukemia diagnosis and treatment offers a promising avenue for advancing the field of nanomedicine. This review has delved into the multifaceted designs of these nanoparticles, highlighting their unique properties and applications that contribute to enhanced diagnostic accuracy and targeted therapeutic interventions. The myriad of innovative approaches, including surface modifications, functionalization, and tailored synthesis methods, underscores the dynamic nature of research in this field. By harnessing the distinctive magnetic properties and biocompatibility of IONPs, researchers have paved the way for improved imaging modalities, early detection, and precise therapeutic delivery in leukemia management. As we navigate the intricate interplay between nanoparticle design and clinical efficacy, it becomes evident that a personalized and targeted approach holds immense potential for transforming leukemia diagnostics and treatment. The amalgamation of cutting-edge materials science, bioengineering, and medical research has propelled the development of IONPs beyond conventional boundaries. However, challenges such as biocompatibility, long-term safety, and scalability remain, necessitating continued collaboration among interdisciplinary experts. Future endeavours should focus on addressing these hurdles to expedite the translation of these promising nanotechnological innovations from bench to bedside. In essence, this review illuminates the captivating journey of IONPs in leukemia research, serving as a testament to the collective efforts driving advancements at the intersection of nanotechnology and medicine. As we stand on the precipice of a new era in leukemia diagnosis and treatment, the evolution of IONPs stands as a beacon of hope, offering a glimpse into a future where precision medicine is not just a concept but a tangible reality.

## Data Availability

No datasets were generated or analysed during the current study.

## Abbreviations

Ab: Antibody
ALL: Acute lymphoblastic leukemia
AMF: Alternating magnetic field
Au: Gold
CD: Cluster of differentiation
CPT: Camptothecin
CTAB: Cetyltrimethylammonium bromide
CTX: Chlorotoxin
DEX: Dextran
DOX: Doxorubicin
EDC/NHS: 1-Ethyl-3-(3-Dimethylaminopropyl)Carbodiimide / N-Hydroxysuccinimide
EpCAM: Epithelial cell adhesion molecule
FA: Folic acid
GNCs: Gold nanoclusters
HL60: Human leukemia-60
IgG1: Immunoglobulin G1
IONPs: Iron oxide nanoparticles
MDA-MB-468: A human breast cancer cell line
MCF-7: Michigan Cancer Foundation-7, A Human Breast Cancer Cell Line
MGMT: O-6-methylguanine-DNA methyltransferase
MoS: Molybdenum disulfide
MXD3: Max dimerization protein 3
NP: Nanoparticle
PAA: Polyacrylic acid
PAP: Polyarginine peptide
PAT: Photoacoustic tomography
PEG: Polyethylene glycol
PEI: Polyethylenimine
PGD: Poly(ethylene glycol) dimethacrylate
PLGA: Poly(lactic-co-glycolic acid)
PVA: Polyvinyl alcohol
siRNA: Small interfering RNA
SORT: Sortilin
SPIONs: Superparamagnetic iron oxide nanoparticles
TEOS: Tetraethyl orthosilicate
TMZ: Temozolomide
TXT: Taxotere
